# Ex vivo propagation of synaptically-evoked cortical depolarizations in a mouse model of Alzheimer’s disease at 20 Hz, 40 Hz, or 83 Hz

**DOI:** 10.1038/s41598-024-74262-2

**Published:** 2024-10-08

**Authors:** Aayushi A. Patel, Mei Hong Zhu, Riqiang Yan, Srdjan D. Antic

**Affiliations:** 1 Department of Neuroscience, School of Medicine, UConn Health, Farmington, CT 06030 USA; 2 Touro College of Osteopathic Medicine, Middletown, NY 10940 USA

**Keywords:** Alzheimer’s disease, High gamma, Excitatory postsynaptic potentials, Cortical layer, Alzheimer's disease, Neural circuits, Excitability

## Abstract

Sensory stimulations at 40 Hz gamma (but not any other frequency), have shown promise in reversing Alzheimer’s disease (AD)-related pathologies. What distinguishes 40 Hz? We hypothesized that stimuli at 40 Hz might summate more efficiently (temporal summation) or propagate more efficiently between cortical layers (vertically), or along cortical laminas (horizontally), compared to inputs at 20 or 83 Hz. To investigate these hypotheses, we used brain slices from AD mouse model animals (5xFAD). Extracellular (synaptic) stimuli were delivered in cortical layer 4 (L4). Leveraging a fluorescent voltage indicator (VSFP) expressed in cortical pyramidal neurons, we simultaneously monitored evoked cortical depolarizations at multiple sites, at 1 kHz sampling frequency. Experimental groups (AD-Female, CTRL-Female, AD-Male, and CTRL-Male) were tested at three stimulation frequencies (20, 40, and 83 Hz). Despite our initial hypothesis, two parameters—temporal summation of voltage waveforms and the strength of propagation through the cortical neuropil—did not reveal any distinct advantage of 40 Hz stimulation. Significant physiological differences between AD and Control mice were found at all stimulation frequencies tested, while the 40 Hz stimulation frequency was not remarkable.

## Introduction

The first signs of cognitive impairment manifest 20–30 years before a clinical AD diagnosis, with synaptic dysfunction occurring prior to significant neuronal loss^1,2^. By the time noticeable cognitive impairments arise, extensive brain areas are already severely affected by amyloid plaques and neurofibrillary tangles^2–4^. These mature plaques and tangles, once formed, are irreversible and cannot be removed from the brain. Therefore, detecting synaptic dysfunction at the initial stages of AD, before these irreversible structural changes, is critical^5,6^. Early intervention could potentially improve synaptic or cognitive functions, offering a window of opportunity to alter the disease progression before severe damage occurs.

Gamma entrainment at 40 Hz has shown promising results in altering the rate of Aβ plaque accumulation and enhancing learning and memory in AD model animals^7–9^. Specifically, combined gamma (40 Hz) pulses of light and sound have been found to protect key brain regions, such as the visual, auditory, hippocampal, and prefrontal cortices of AD model animals^9^. Histological and transcriptomics data indicate that chronic gamma stimulation helps shift cortical neurons to a less degenerative state and improves synaptic function^8^. Notably, invasive artificial driving of cortical circuits at gamma (40 Hz), but not at other frequencies, recruits neuronal and glial responses to reduce Aβ levels in the visual cortex^7^. The unique effectiveness of the 40 Hz frequency raises the question: What makes 40 Hz so special? We hypothesize that stimuli at 40 Hz propagate more efficiently both between cortical layers (vertically) and along cortical laminas (horizontally) compared to inputs at 20 Hz or 83 Hz. At the 40 Hz gamma frequency, subthreshold synaptic events may optimally undergo spatiotemporal summation in the cortical circuitry.

To test this hypothesis, we utilized a population voltage imaging method^10^. Multi-site optical imaging of population membrane potential changes employing genetically-encoded voltage indicators (GEVIs)^11,12^ is uniquely suited for monitoring subthreshold synaptic potentials in populations of neurons belonging to specific cell types^13,14^. Measurements conducted simultaneously in several cortical layers^10^ provide a robust tool for quick and thorough assessment of evoked network responses in *Control* (Healthy) and *Test* (AD) animals^15^. Comparisons of synaptic inputs were made between the AD Group (5xFAD) and their healthy littermates (Control Group) using the "medium-gamma" (40 Hz) frequency, as well as two other frequencies: "below-gamma" (20 Hz) and "high-gamma" (83 Hz). Our aim was to precisely understand how a 40 Hz medium-gamma input propagates between two cortical layers vertically or two cortical columns horizontally. Additionally, we sought to contrast the 40 Hz data with the lower (20 Hz) and higher frequency (83 Hz) data. Finally, our goal was to identify any changes in summation or propagation of cortical depolarizations attributable to the presence of ongoing AD Aβ-mediated pathology.

## Methods

### Ethics information

This study is reported in accordance with ARRIVE guidelines. All methods were performed in accordance with the relevant guidelines and regulations. The experimental methods were approved by the UConn Health Institutional Animal Care and Use Committee under animal protocol #AP-200902. Every effort was made to reduce the number of animals used, and to minimize their suffering.

### Animals

**Fig. 1:**
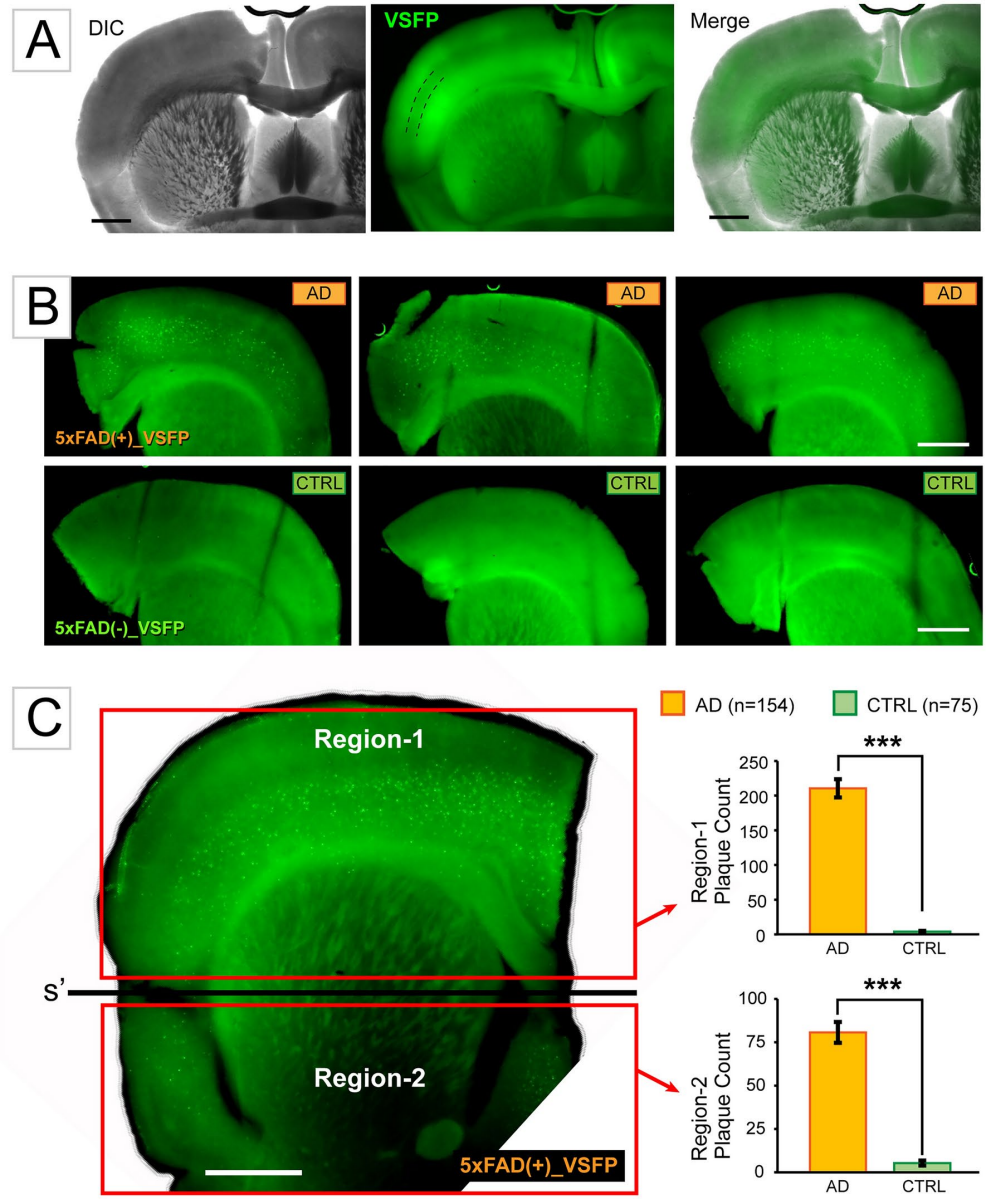
Amyloid plaques in the 5xFAD-GEVI animal model. (**A**) Transgenic animal line expressing a GEVI variant called “*VSFP*” in all pyramidal neurons. Scale, 1 mm. (**B**) Brain sections from two experimental groups displayed in 2 rows. Top row: The “*AD Group*” combines the 5xFAD mouse model of AD with GEVI expression. Bottom row: The “*CTRL Group*” consists of healthy littermates who did not inherit the 5xFAD genes, but did inherit the GEVI genes for voltage imaging. Scale, 1 mm. (**C**) Amyloid plaques were stained with thioflavin-S and counted in two arbitrary regions, separated by a horizontal line, *s’*, transversing the striatum. *Region-1:* Superior cortex. *Region-2:* Inferior cortex. Scale, 1 mm.

Alzheimer’s Diseased mice, 5xFAD, obtained from The Jackson Laboratory (MMRRC Strain #034840-JAX), were bred with mice expressing a genetically encoded voltage indicator (VSFP), obtained from the Thomas Knopfel Laboratory (CaMK2A-tTA;tetO-chiVSFP). This breeding produced double transgenic mice (AD + and GEVI +), as well as single transgenic mice (GEVI + alone), establishing our experimental groups. Offspring positive for both the AD and GEVI genes were labeled as “AD-GEVI’' or the “AD Group’'. Offspring positive only for GEVI were designated as the “Control Group’' (Fig. 1A, B). All animals were housed in standard conditions with unrestricted access to food and water, under a 50% dark/light cycle. Brain slices were obtained from transgenic mice (aged 90–130 days, of both sexes), during the light phase of the circadian cycle, between 10:00 a.m. and Noon.

### Wide-field voltage imaging

Animals were sacrificed in deep halothane anesthesia, and their brains were extracted into an ice-cold saline solution consisting of (in mM): 125 NaCl, 26 NaHCO3, 2.3 KCl, 1.26 KH2PO4, 2 CaCl2, 1 MgSO4, and 10 glucose. Coronal slices from the frontoparietal cortex were prepared using a Leica VT1000 vibrating blade microtome. These slices were incubated at 37 °C for 30 min, then cooled at room temperature. The brain slices were transferred to an Olympus BX51WI upright microscope and perfused with aerated saline containing 5% CO_2_ and 95% O_2_. All experimental measurements were conducted at 34 °C. Stimulation electrodes, fabricated from 1.5 mm borosilicate glass with a filament (resistance ∼2 MΩ), and backfilled with saline, were positioned in cortical layer 4 (L4). Extracellular stimulation was administered through a stimulus isolation unit (IsoFlex, A.M.P.I., Israel), employing a fixed current intensity of 135 nA. Optical signals were recorded simultaneously from three regions of interest (ROIs). ROI 1 in L4, and ROI 2 and ROI 3 in L2/3. Synaptic stimulation trains of 20 Hz, 40 Hz, and 83 Hz were tested within the same brain slice. Optical trials typically involved 3 s of light exposure with at least 12-s intervals between consecutive sweeps. Optical signals were sampled at a full-frame interval of 1.020 ms (∼1 kHz frame rate) using a NeuroCCD camera (80 × 80 pixel; RedShirtImaging, Decatur, GA). The genetically encoded voltage indicator (GEVI, VSFP) was excited using a 470 nm light-emitting diode (LED) (pE, CoolLED, Andover, UK) and imaged with a filter set comprising excitation (480/40 nm), dichroic (510 nm), and emission (535/50 nm) filters. The GEVI (VSFP) signals recorded using the green emission filter (535/50 nm) exhibited a negative polarity in raw data records. However, for clarity, these optical signals have been inverted in the figures. We believe that displaying inverted GEVI optical signals (positive with depolarization) is more suitable for visual comprehension.

### Still imaging and plaque counting

At the conclusion of the voltage-imaging sessions, brain slices were removed from the recording chamber and fixed in 4% paraformaldehyde. The slices were then incubated in a 1% aqueous solution of Thioflavin-S, mounted on microscope slides, and photographed using a Keyence fluorescence microscope BZ-X800 equipped with a 2 × dry objective. Each 300 μm thick brain slice was examined on both its top and bottom side, and both surfaces were included in the analysis. Thioflavin-S-stained plaques were counted manually in two brain slice areas: Region-1, comprising superior, and Region-2, comprising the inferior cortex (Fig. 1C). The individuals performing the counting were blinded to the experimental group of the brain slices.

### Data analysis

The optical traces were processed and analyzed using Neuroplex software (RedShirtImaging, LLC). Bleach correction was performed by subtracting an exponential fit from each optical trace. Spatial averaging and filtering were applied post-hoc, during data quantification, not during the recordings.

#### Temporal summation

To enhance the signal-to-noise ratio for assessing temporal synaptic summation, each trace underwent temporal averaging (n = 4 sweeps), spatial averaging (57 pixels), and filtering with a low-pass Gaussian filter with a 33 Hz cutoff, without a high-pass filter. Temporal summation was determined by calculating the ratio of the amplitudes of the second peak (Peak-2) to the first peak (Peak-1) within the same optical trial.

#### Spatial propagation

For quantifying the propagation strength of evoked cortical depolarizations, each trace underwent temporal averaging (n = 4 sweeps), spatial averaging (37 pixels), and filtering with a low-pass Gaussian filter (77 Hz cutoff), and a high-pass RC filter (0.1 Hz cut off). Spatial propagation efficacy (strength) was calculated as the ratio of the peak amplitude at a remote location to the peak amplitude at the stimulation site, within the same optical trial.

Quantitative analyses were organized using Excel. Results are presented as mean ± standard error of the mean (SEM), unless otherwise indicated. Statistical significance was assessed in GraphPad Prism using a one-way ANOVA (*p* ≤ 0.05) followed by multiple comparisons with a Tukey’s test. The four levels of statistical significance (0.05; 0.01; 0.001; and 0.0001) were denoted in figure panels by one, two, three or four asterisks, respectively.

## Results

### Utilizing the AD-GEVI mouse model

Two mouse lines, namely the AD line (5xFAD) and the GEVI line (CaMK2A-tTA;tetO-chiVSFP) were crossed, resulting in the “5xFAD-GEVI” mouse line, which combines an AD pathology with the GEVI expression in pyramidal cells. The GEVI expression is controlled by the CaMK2A promoter and tetracycline response element (TRE), achieving three key features: (i) dense, (ii) cell-type-specific (specifically in neocortical excitatory pyramidal cells), and (iii) cortex-wide expression of the GEVI (Fig. 1A), see also^10,16–18^.

In the visualization of GEVI expression, the VSFP was observed in all cortical regions, except for the inferior temporal cortex (Fig. 1A, Merge). VSFP was expressed strongly in all cortical layers, except L4. At cortical layer L4, we constantly observed a darker band (Fig. 1A, dashed lines), reflecting a lower density of fluorescently-labeled pyramidal cells in this lamina. Around 90 days after birth (postnatal 90, P90), the AD-GEVI mice accumulated amyloid plaques, as revealed by thioflavin-s staining (Fig. 1B, the AD row). In contrast, the age-matched healthy littermates within the range P90–P132 did not exhibit plaques in the brain (Fig. 1B, the CTRL row).

Thioflavin-s-positive plaques were counted in two cortical regions: *Region-1*, covering the superior cortex, and *Region-2*, encompassing the inferior cortex (Fig. 1C). A delineation between these two counting regions was established by employing an arbitrary line through the striatum (Fig. 1C, black horizontal bar, ***s’***). In the AD Group, we analyzed 154 images from 77 thioflavin-s-treated brain slices obtained from thirty-three 5xFAD-positive & VSFP-positive animals. Conversely, in the CTRL Group, we analyzed 75 pictures from 38 thioflavin-s-treated brain slices obtained from nineteen 5xFAD-negative & VSFP-positive animals. In Region-1 of the AD group, the plaque count was (mean ± sem) 210.5 ± 13.1 plaques (n = 154 images). In contrast, the CTRL group exhibited only 4.0 ± 0.9 plaque-like spots per area in Region-1 (n = 75 images). The plaque load in cortical Region-1 was found to be significantly different between the AD and CTRL groups, with a *p* value less than 0.00001. Moving to Region-2, the AD group displayed a plaque count of 80.7 ± 6.0 plaques per area (n = 154 images), while the CTRL group showed only 5.4 ± 1.45 plaques in Region-2 (n = 75). Similar to Region-1, the plaque load in cortical Region-2 was significantly different between the AD and CTRL groups, with a *p* value less than 0.00001 (Fig. 1C, bar graphs).

### Multi-site voltage imaging of evoked cortical depolarizations

**Fig. 2:**
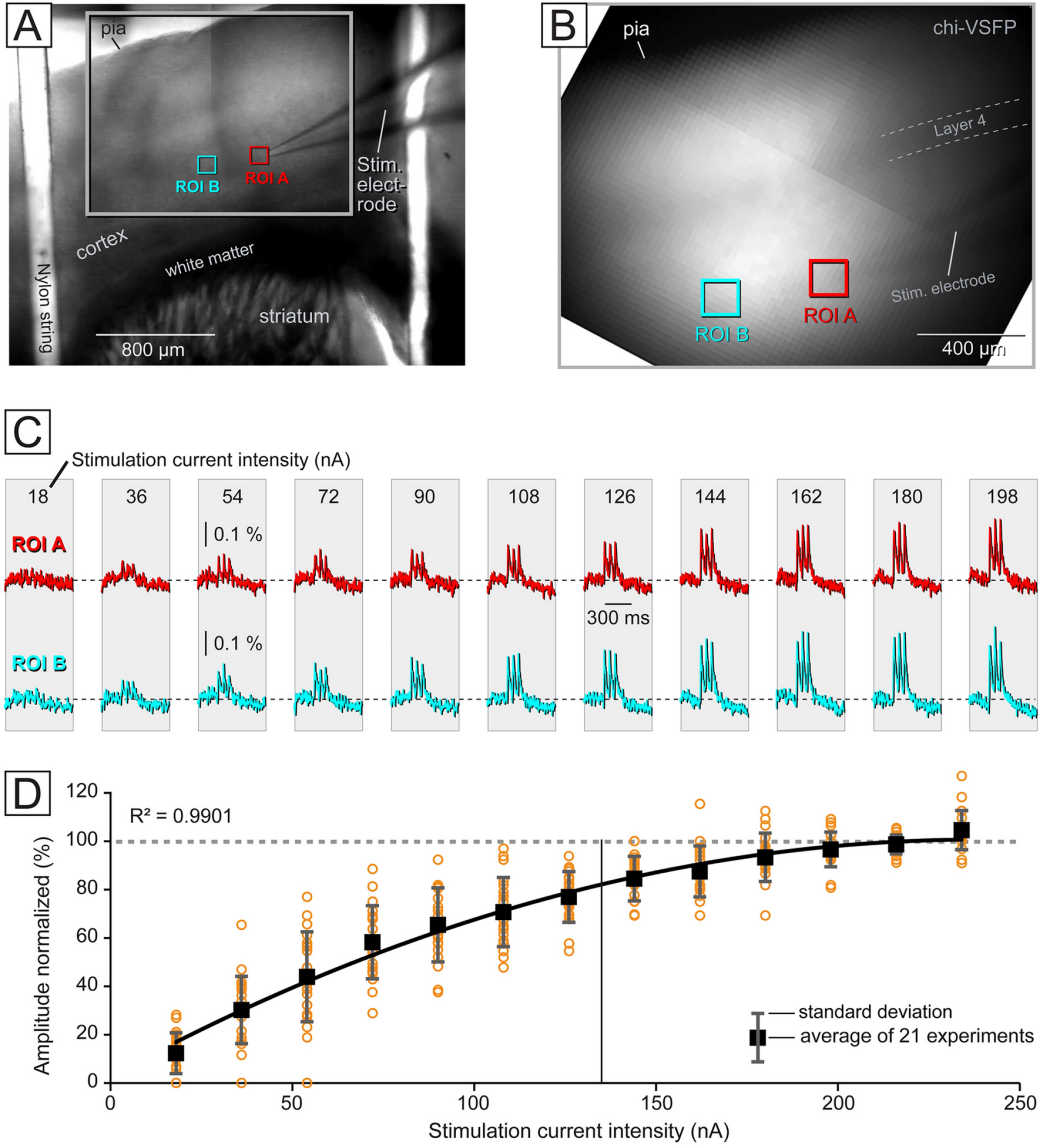
Gradual increase in stimulation intensity. (**A**) Brain slice from a 5xFAD-GEVI animal observed inside a recording chamber, captured with a 4× lens. (**B**) Surface of a brain slice imaged with a 10 × lens. The extracellular stimulation electrode is positioned in ROI-A. (**C**) Synaptically-evoked cortical depolarizations recorded simultaneously at two regions of interest (ROI-A and ROI-B). Eleven experimental trials are displayed, with the stimulation current intensity (indicated at the top) gradually increasing from 18 to 198 nA across successive trials. (**D**) Sequences of gradually increasing stimulation intensities (13 trials) were obtained in 21 locations of 6 brain slices, from 3 animals. Each black rectangle represents the average of 21 data points (orange circles). The trendline shows a polynomial fit, with the vertical line marking the “135 nA stimulation current intensity”.

Before examining the physiological distinctions between two experimental groups, labeled as AD and CTRL, our initial objective was to determine the optimal intensity of the external stimulus. Brain slices were positioned in the recording chamber of an upright microscope equipped with a voltage imaging camera, and a glass stimulation electrode was positioned to contact the tissue surface (Fig. 2A, B). To elicit cortical depolarizations (Fig. 2C), three current pulses with a 120 ms interstimulus interval (8.3 Hz) were administered. The current intensity was methodically increased in uniform increments. For each current intensity level, we conducted four optical trials, which were subsequently employed for temporal averaging. Consistently, twelve to thirteen consecutive experimental trials were conducted at the same location. In the exploration of six brain slices from 3 animals, we investigated a total of 21 locations. The resulting plot in Fig. 2D depicts the optical signal amplitude in relation to the stimulus current intensity. At the midpoint of the current intensity range (indicated by the vertical black line in Fig. 2D), we selected a specific intensity (135 nA) for use in subsequent experiments. Lower current intensities did not produce sufficiently strong optical signals, while higher current intensities did not yield any major improvement in the optical signal amplitude. Rather, the response amplitude appeared to saturate at high intensities, suggesting a near complete recruitment of excitable elements at the stimulation site (Fig. 2D).

### Investigating cortical responses to high-frequency stimulation

**Fig. 3:**
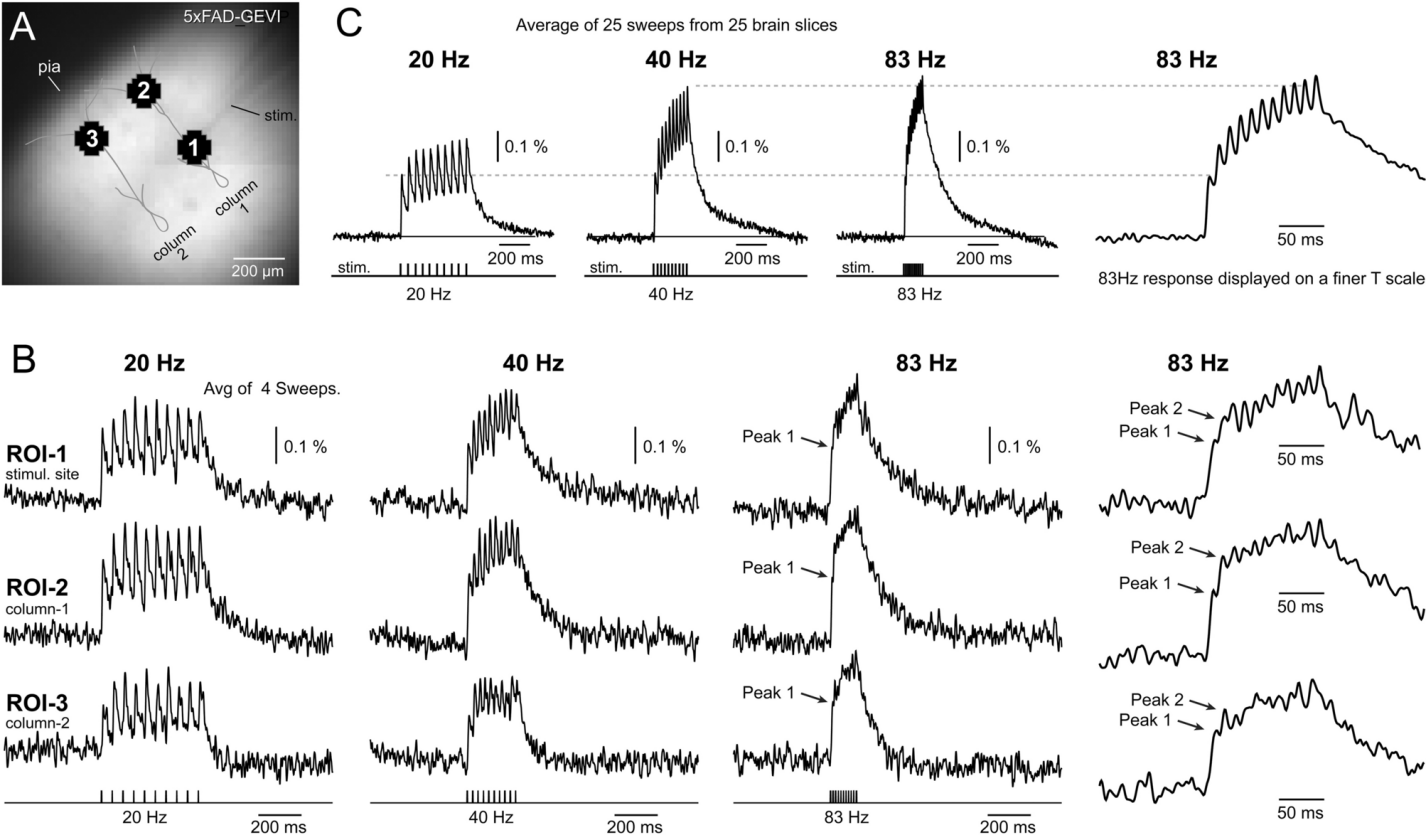
High Frequency Stimulation. (**A**) Image of a brain slice from a 5xFAD-GEVI mouse, projected onto an 80 × 80-pixel voltage-imaging camera. The extracellular stimulation electrode is placed in layer-4. The drawings depict pyramidal cells in two neighboring cortical columns. (**B**) Ten stimulation pulses were delivered at three different frequencies: 20 Hz, 40 Hz, and 83 Hz. Evoked cortical depolarizations were optically recorded in three regions of interest (ROIs 1-3) as marked in (*A)*. Each trace is filtered with a 33 Hz low-pass filter, and represents an average of four experimental trials from the same brain slice. At 83 Hz stimulation frequency, the first event (*Peak 1*) is discernible on the rising phase of the optical voltage transient (arrows). On a finer time scale, the individual peaks at 83 Hz are less distinct compared to the peaks obtained in the same brain slice, but at lower frequency (20 Hz & 40 Hz). (**C**) Each trace represents an average of 25 brain slices. Averaging enhances the quality of the optical voltage signals, revealing finer details in the waveforms. The lower dashed horizontal line indicates *Peak 1*, the cortical response to the first stimulus in a train. The upper dashed horizontal line marks the maximum peak at 40 Hz (*medium-gamma frequency)*. The cortical depolarization evoked by 83 Hz stimulation is also shown on a faster time scale to resolve individual peaks.

In subsequent series of experiments, the stimulation electrode was consistently positioned in L4 (Fig. 3A), where afferent sensory inputs predominantly impinge onto the cerebral cortex. To assess cortical responses to high-frequency stimulation, 10-pulse trains were administered at 20, 40, and 83 Hz, and the resulting optical signals were recorded at three distinct locations, denoted as 3 ROIs (Fig. 3B). ROI-1 was chosen within layer L4, precisely at the stimulation site (stim.), while ROI-2 and ROI-3 were designated within cortical layer 2/3 (Fig. 3A). Notably, ROI-2 always occupied the same cortical column as the stimulus, while ROI-3 was positioned in the adjacent cortical column, approximately 300 µm away from ROI-2. This configuration of ROIs was employed for evaluating the strength of signal propagation. Vertically, we gauged the efficiency of propagation from L4 to L2/3 by computing the amplitude ratio ROI-2/ROI-1 (“ROI 2/1”). Horizontally, we quantified the efficacy of electrical signal propagation using the amplitude ratio ROI-3/ROI-2 (“ROI 3/2”).

The optical detector (Neuro-CCD) boasted a relatively high sampling rate of 1 kHz, coupled with a relatively good optical signal quality, attributed to the robust expression of VSFP in all pyramidal neurons (Fig. 1). This combination enabled us to discern individual cortical responses to each stimulus pulse at 20 Hz (interstimulus interval 50 ms) and 40 Hz (interstimulus interval 25 ms) (Fig. 3B). However, at the stimulation frequency of 83 Hz (interstimulus interval 12 ms), the voltage transients coalesced into a complex optical waveform (Fig. 3B, 83 Hz). This fast stimulation frequency produced partially summed population responses, where individual peaks became less distinguishable due to several factors: (i) temporal jitter, (ii) trial-to-trial variation in EPSP success, and most critically, (iii) low signal-to-noise ratio (SNR). Nevertheless, the rising phase of the 83 Hz signal still revealed the first peak (Peak 1), and on expanded traces, the second peak (Peak 2) could also be identified (Fig. 3B, far right traces).

Employing data from multiple brain slices (n = 25), all obtained with an identical stimulation paradigm (stimulus pattern), and identical optical sampling rate (1 kHz), we conducted temporal alignment of optical traces and subsequent averaging. This procedure improves the SNR by the square root of the number of traces averaged. In this example, SNR was improved by 5 (√n) compared to optical data from one trace alone. Averaged voltage waveforms (Fig. 3C) may represent an entire experimental group with 25 or more brain slices (e.g., AD-Male or CTRL-Male), facilitating the identification of differences between the AD and CTRL cohorts based on the features of the voltage waveforms.

### Temporal summation

**Fig. 4:**
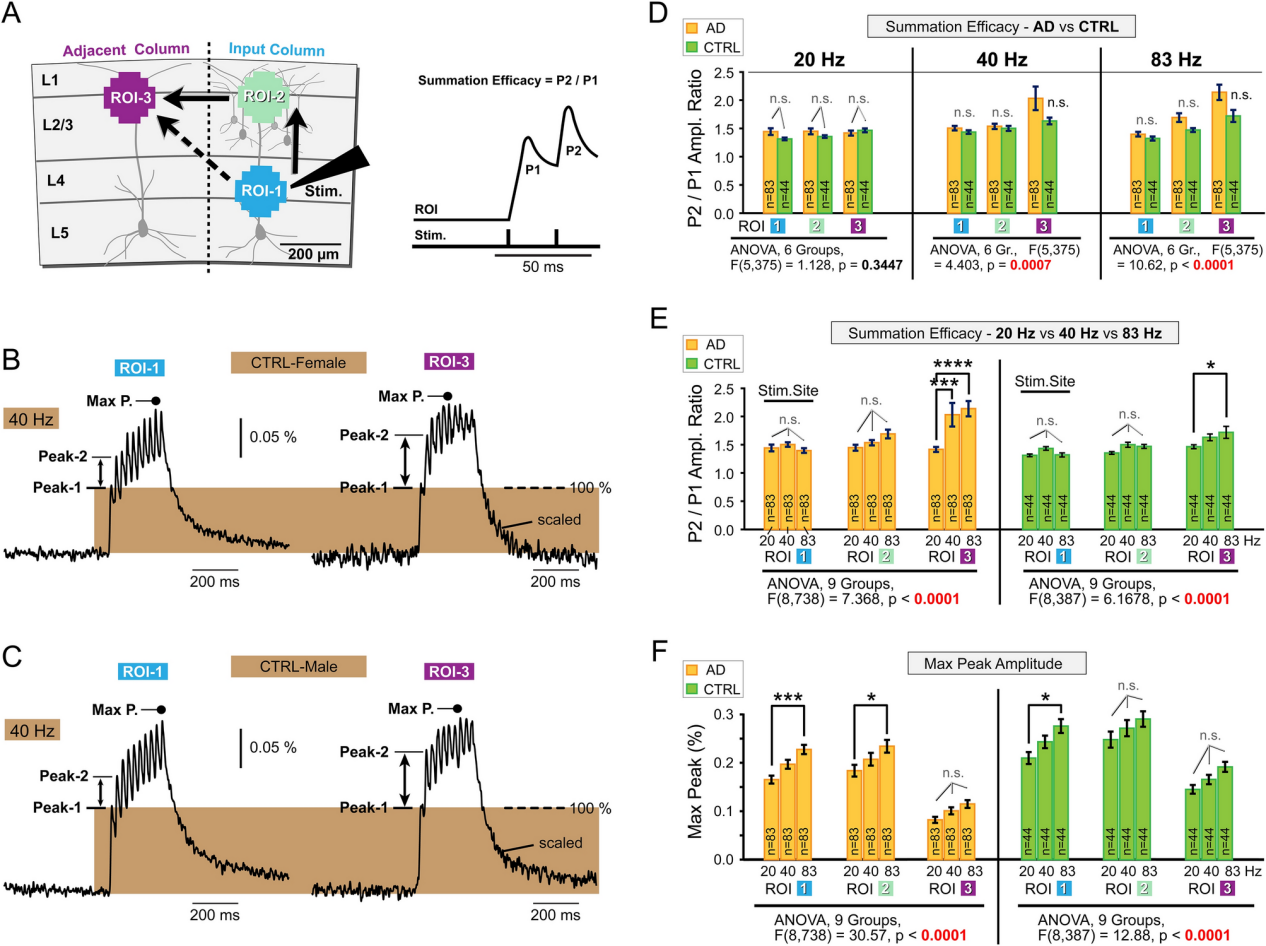
Temporal Summation Efficacy. (**A**) Left: Experimental outline. The stimulation electrode is positioned in layer 4 (L4). Electrical signals may propagate between three ROIs. Black uninterrupted arrows indicate “canonical propagation”, while the dashed arrow is a “non-canonical propagation”. Right: Synaptic summation efficacy is defined as the amplitude ratio of Peak-2 to Peak-1 (P2/P1). (**B**) Cortical response at ROI-1 (left) and ROI-3 (right), upon ten stimulation pulses at 40 Hz. The optical signal amplitude at ROI-3 is scaled to match Peak-1 (P1) in ROI-1 (e.g., normalized to P1). The height of the brown rectangle is set to 100% of Peak-1 to illustrate the relative amplitude gain in Peak-2. (**C**) Same as in (**B**), except in the CTRL-Male experimental group. (**D**) Bar graphs represent average P2/P1 amplitude ratios per ROI for each experimental group, at three stimulation frequencies: 20, 40, and 83 Hz. Each bar represents an average of 83 slices in the AD group (orange) and 44 brain slices in the CTRL group (green). Data from both sexes are combined: AD-Female (n = 42 slices, from 14 mice), AD-Male (n = 41 slices, from 12 mice), CTRL-Female (n = 18 slices, from 7 mice), and CTRL-Male (n = 26 slices, from 8 mice). One-way ANOVA indicated that null hypothesis is not rejected for 20 Hz, but rejected for the 40 Hz and 83 Hz data. Post-hoc Tukey’s tests did not detect any significant differences between bars belonging to the same stimulation frequency. "n.s."—not significant. (E) Same data as in (*D*), organized to test the effect of the stimulation frequency on the summation efficacy. One-way ANOVA followed by post-hoc Tukey’s tests detected significant differences between three stimulation frequencies only at the most remote recording site (ROI-3) in both experimental cohorts, AD animals (Left, orange) and CTRL animals (Right, green). The summation efficacy, expressed as the P2/P1 ratio, is significantly stronger during 83 Hz versus 20 Hz synaptic stimulation (asterisks). [*] *p* < 0.05; [**] *p* < 0.01; [***] *p* < 0.001; [****] *p* < 0.0001. (F) Bar graphs represent the average maximal Peak (“*Max P*”) as labeled in panels (*B*) and (*C*). A relatively consistent finding is that the maximal optical signal amplitude achieved during a 10-pulse train (“*Max P*”) was significantly higher at 83 Hz stimulation compared to the 20 Hz stimulation frequency. Note that the average Max Peak amplitude of the evoked cortical depolarization increases gradually with the stimulation frequency: 20 Hz < 40 Hz < 83 Hz, regardless of the experimental group (AD, orange or CTRL, green); and regardless of the recording site (ROI-1, ROI-2 or ROI-3).

In this series of ex-vivo recordings, a total of 26 AD mice and 15 CTRL mice were utilized. The age range of the animals spanned from 90 to 132 days. Voltage imaging experiments were conducted on 2–4 brain slices from the same animal, with standardized extracellular stimulation using an electrode inserted into L4 (Fig. 4A) and a fixed stimulation current intensity of 135 nA (Fig. 2). Each brain slice (83 AD and 44 CTRL) received trains of 10 stimulation pulses at three different frequencies: 20, 40, and 83 Hz. We placed ROI-1 in L4, and ROI-2 in L2/3 of the same cortical column (Fig. 4A), following the canonical cortical circuit model, which posits predominant signal propagation from the input layer (L4) into L2/3 (Fig. 4A, vertical full arrow). Additionally, ROI-3 was included in the neighboring column (Fig. 4A, ROI-3) to monitor the spread of compound depolarizations (population signals) through the superficial cortical layers (L1/2/3), from ROI-2 to ROI-3 (horizontal full arrow). In the experimental diagram, we also include a hypothetical non-canonical propagation of electrical signals through cortical neuropil from the stimulation site of the input column (ROI-1) to L2/3 of the adjacent cortical column, ROI-3 (Fig. 4A, oblique dashed arrow). At each recording location (ROI—region of interest), we evaluated a paired pulse ratio (Peak-2 (P2) divided by Peak-1 (P1)), referred to as the "*summation efficacy*" of compound synaptic depolarizations (Fig. 4A, right). A higher P2/P1 ratio indicates greater summation efficacy. In Fig. 4B, C, we illustrate a method for direct comparison of *summation efficacies* directly from experimental traces (for example, ROI-1 vs ROI-3 in the same brain slice). To illustrate the “summation efficacy”, the amplitude of Peak-1 in ROI-3 was scaled using the amplitude of Peak-1 in ROI-1 of the same brain slice. Therefore, the rectangular brown area represents 100% of Peak-1 in both locations (Fig. 4B). Peak-2 will rise above this 100%-line based on the “*summation efficacy*”. In two examples obtained from two experimental groups (Fig. 4B, C), the summation efficacy was greater in ROI-3 than in ROI-1. This is likely due to the origin of optical signal in two locations. In ROI-1, at the stimulation site, there is a stronger influence of action potentials (APs) caused by the stimulation electrode (Stim.). Being all-or-none potentials, APs do not summate well in the neuronal membrane. Away from the stimulation site, at ROI-3, optical signals are more dependent on EPSPs. EPSPs do summate in neuronal membranes, hence stronger synaptic summation in ROI-3.

In the numerical analysis (Fig. 4D), we asked if *summation efficacy* (P2/P1 ratio) is affected by the presence of AD genes. The AD group is represented by 83 measurements—83 brain slices from twenty-six 5xFAD-positive animals: combined sexes (12 male and 14 female AD mice). The Control group comprises 44 recordings in 44 brain slices obtained from 15 healthy littermates (fifteen 5xFAD-negative animals), combined sexes (8 male and 7 female AD mice). The recordings were quantified at three ROIs (ROI-1, ROI-2, and ROI-3). The multisite voltage-imaging approach allowed us to explore potential AD gene-mediated changes at different cortical layers (L4 and L2/3), at two neighboring cortical columns (input column and adjacent column, Fig. 4A). Most importantly, all measurements in all brain slices (n = 127) were performed at three stimulation frequencies (20, 40 and 83 Hz).

In Fig. 4D, data groups were organized based on stimulation frequency and ROI. At 20 Hz, no significant differences were observed between groups (ANOVA, *p* > 0.05). The results of the ANOVA test are presented below the corresponding bar graph. At 40 Hz and 83 Hz, one-way ANOVA indicated significant differences among the data bars (*p* < 0.001). However, post-hoc Tukey’s tests did not reveal statistically significant differences between the matching AD and CTRL data (Fig. 4D, “n.s.”). Here, “matching” refers to data obtained at the same distance from the stimulation site, within the same ROI type (ROI-1, 2 or 3). Since the recording site (ROI) influences optical signal amplitudes (Fig. 3), comparisons between data sets within the same ROI are the most valid for AD-to-CTRL comparisons. In conclusion, temporal summation efficacy of compound EPSPs, expressed as the P2/P1 ratio, did not distinguish AD from CTRL brain slices. The P2/P1 amplitude ratio failed to predict the animal condition (AD vs. CTRL) across all tested stimulation frequencies (20, 40, and 83 Hz, Fig. 4D). Notably, the 40 Hz stimulation frequency did not exhibit any unique characteristics.

In Fig. 4E, we investigated whether stimulation frequency influences the efficacy of temporal summation. To eliminate the confounding effects of genotype, the AD group (orange bars) and the CTRL group (green bars) were analyzed separately. Notably, the same brain slice was sequentially stimulated at 20 Hz, 40 Hz, and 83 Hz in these experiments, minimizing variability between slices. ANOVA analysis of traces from the same brain slice and ROI across the three stimulation frequencies revealed that frequency had a significant overall effect on the P2/P1 ratio (ANOVA, *p* < 0.001). Pairwise multiple comparisons (Tukey’s tests) indicated that 83 Hz stimulation produced greater summation efficacy (higher P2/P1 ratio) than 20 Hz, but this effect was only observed at one of the three ROIs tested (Fig. 4E, asterisks). Specifically, the significant effect of stimulation frequency on temporal summation strength was confined to ROI-3, the most distal recording site. Interestingly, this pattern was consistent across both the AD and CTRL groups (Fig. 4E), enhancing confidence in the population voltage imaging data and the resulting conclusions.

Based on intracellular recordings from individual neurons, EPSP summation efficacy is expected to increase progressively with higher stimulation frequencies^19^. We observed this trend (“20 < 40 < 83 Hz”) at ROI-3, where synaptic potentials likely dominate the optical signal. However, at the stimulation site (ROI-1), the average P2/P1 ratio did not follow the expected orderly increase with stimulation frequency—83 Hz did not elicit a stronger response than 20 Hz or 40 Hz in either the AD or CTRL group (Fig. 4E, Stim. Site). This suggests that at ROI-1, action potentials are numerous and do not summate effectively. In summary, our ex vivo population voltage imaging data (Figs. 2, 3, 4) are consistent with findings from extracellular (LFP) and intracellular (whole-cell) recordings of synaptic summation in the cerebral cortex^19,20^. Summation efficacy increases with stimulation frequency, with the most significant differences observed at recording sites farthest from the stimulation electrode. At 40 Hz and 83 Hz stimulation frequencies, temporal summation (paired pulse ratio, P2/P1) was significantly stronger at the most distal site (ROI-3) compared to the stimulation site (ROI-1) (Tukey’s test, *p* < 0.001) (Suppl. Fig. S1).

In Fig. 4F, we quantify maximal amplitude of the optical signal (*Max Peak*) reached during the 10-pulse stimulation train (Fig. 4B , C “*Max P*”). Unlike the P2/P1 ratio discussed in Fig. 4E, the hierarchy of 20 < 40 < 83 Hz is more clearly established in Fig. 4F, with the *Max Peak* at 83 Hz being significantly greater than at 20 Hz (Fig. 4F, blue asterisks). Notably, during the 10-pulse train of synaptic depolarizations, each ROI analyzed in this study exhibits greater maximal depolarization at higher frequencies, following the pattern 83 > 40 > 20 Hz.

### Spatial propagation of evoked cortical depolarizations

**Fig. 5:**
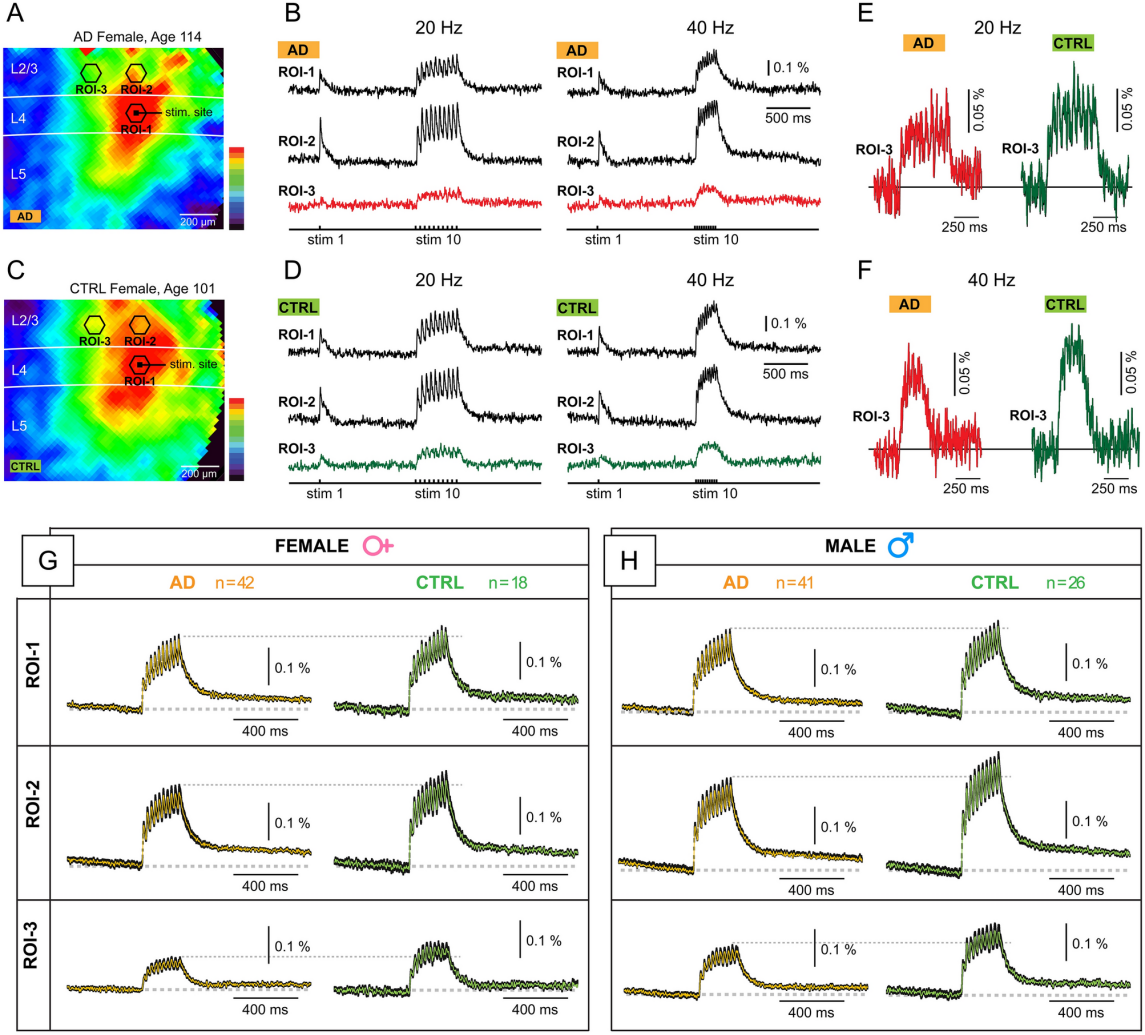
Weaker amplitude—similar voltage waveforms. (**A**) Female animal (age 114 days) in the AD experimental group (5xFAD-positive). Voltage imaging of synaptic depolarizations evoked in L4. The image is a result of frame subtraction, representing the difference between the peak of the optical signal and the baseline before the stimulus. Optical signal amplitude at each pixel is color-coded, with red indicating maximal, and dark-blue color indicating minimal amplitude. (**B**) The extracellular stimulation paradigm consisted of one pulse (stim 1) followed by a train of 10 pulses (stim 10). Three regions of interest (ROIs) are selected for trace-display (amplitude vs. time). Two consecutive experimental trials are shown: 20 Hz stimulation frequency (left), and 40 Hz (right). (**C** and** D**) Same as (A) and (B) for a different animal—Female (age 101) from the CTRL group (5xFAD-negative). (**E**). Comparison of traces obtained at ROI-3 between AD and CTRL animals, at 20 Hz stimulation frequency. (**F**) Same as (E), but at 40 Hz stimulation frequency. (**G** and** H**) Each colored trace represents a group average (mean). “n” indicates the number of slices averaged per group. The thickness of the black trace represents the standard error of the mean (s.e.m.). Dashed horizontal gray lines are drawn for quick comparison between sex-matched AD group (orange) vs CTRL group (green).

In the analysis of the spatial propagation efficacy, all brain slices (underwent identical stimulation procedures, with an extracellular stimulation electrode inserted into L4, Fig. 5A, C). Each brain slice (n = 127) received trains of 10 stimulation pulses at three frequencies, 20, 40 and 83 Hz (*stim 10*). We performed spatial averaging of pixels captured inside a given ROI (37 pixels per ROI), so that each ROI is represented by just one optical signal (Fig. 5B, D). Preceding the 10-pulse-train, a single stimulation pulse was delivered (*stim 1*), producing detectable cortical depolarizations at the stimulation site (ROI-1), or in the same cortical column (ROI-2) (Fig. 5B, D). Using a consistent color-coded scaling of optical signal amplitudes, we presented the amplitude achieved at each pixel, at a specific time point (Fig. 5A, C). In this example, we choose a time point at which the first response in the 10-pulse-train reached its peak (Fig. 5B, D). Color-coded voltage maps indicated a comparatively weaker spatial spread of synaptically-evoked cortical depolarizations in the AD animals (Fig. 5A) as opposed to CTRL animals (Fig. 5C). At a distant recording site, ROI-3, the optical signal amplitude was often smaller in AD compared to CTRL animals, regardless of the frequency of the stimulus train, such as 20 Hz (Fig. 5E); or 40 Hz (Fig. 5F, compare red versus green trace); or 83 Hz (not shown).

Our experimental design for testing physiological differences between AD and CTRL conditions included 4 experimental groups (AD-Female, CTRL-Female, AD-Male, and CTRL-Male). More specifically, the AD-Female comprised 42 slices, from 14 mice. The AD-Male group contained 41 slices, from 12 mice. The CTRL-Female included 18 slices, from 7 mice, and CTRL-Male group encompassed 26 slices, from 8 mice. To generate voltage waveforms representative of each experimental group, we averaged optical traces obtained from all brain slices belonging to a given experimental group. All optical traces were aligned based on the stimulus pulses (stim.). In Fig. 5G, a colored trace (orange or green) represents the group mean at each sample point, while the black trace represents the standard error of the mean (s.e.m.). Similar to the individual voltage maps (Fig. 5A, C), in the group average, again the AD condition (orange) appeared to exhibit weaker propagation than the CTRL condition (green), manifested by smaller amplitudes at both ROI-2 and ROI-3 (Fig. 5G, H) compare AD vs CTRL). These data suggest that in both female (Fig. 5G) and male (Fig. 5H) mice, the presence of AD genes (AD experimental group) reduced the amplitude of optically-recorded evoked cortical depolarization, on average (note that each trace is an average of the entire experimental group). However, apart from amplitude being stronger in CTRL, the averaged traces did not reveal obvious differences in other parameters of voltage waveforms (rise, duration, decay) obtained either at the stimulation site in L4 (ROI-1), in layer 2/3 of the input column (ROI-2), or in layer 2/3 of the adjacent cortical column (ROI-3). That is, direct visual comparison of orange (AD) and green (CTRL) traces did not reveal any systematic differences caused by the presence of the AD genes (5xFAD). In the next section, instead of averaging traces, we will quantify each experiment individually, calculate ratios (e.g., ROI-2 / ROI-1) for each brain slice, and then perform statistical comparisons between experimental groups (AD-Female, CTRL-Female, AD-Male, and CTRL-Male).

### Strength of propagation in the AD-female group

**Fig. 6:**
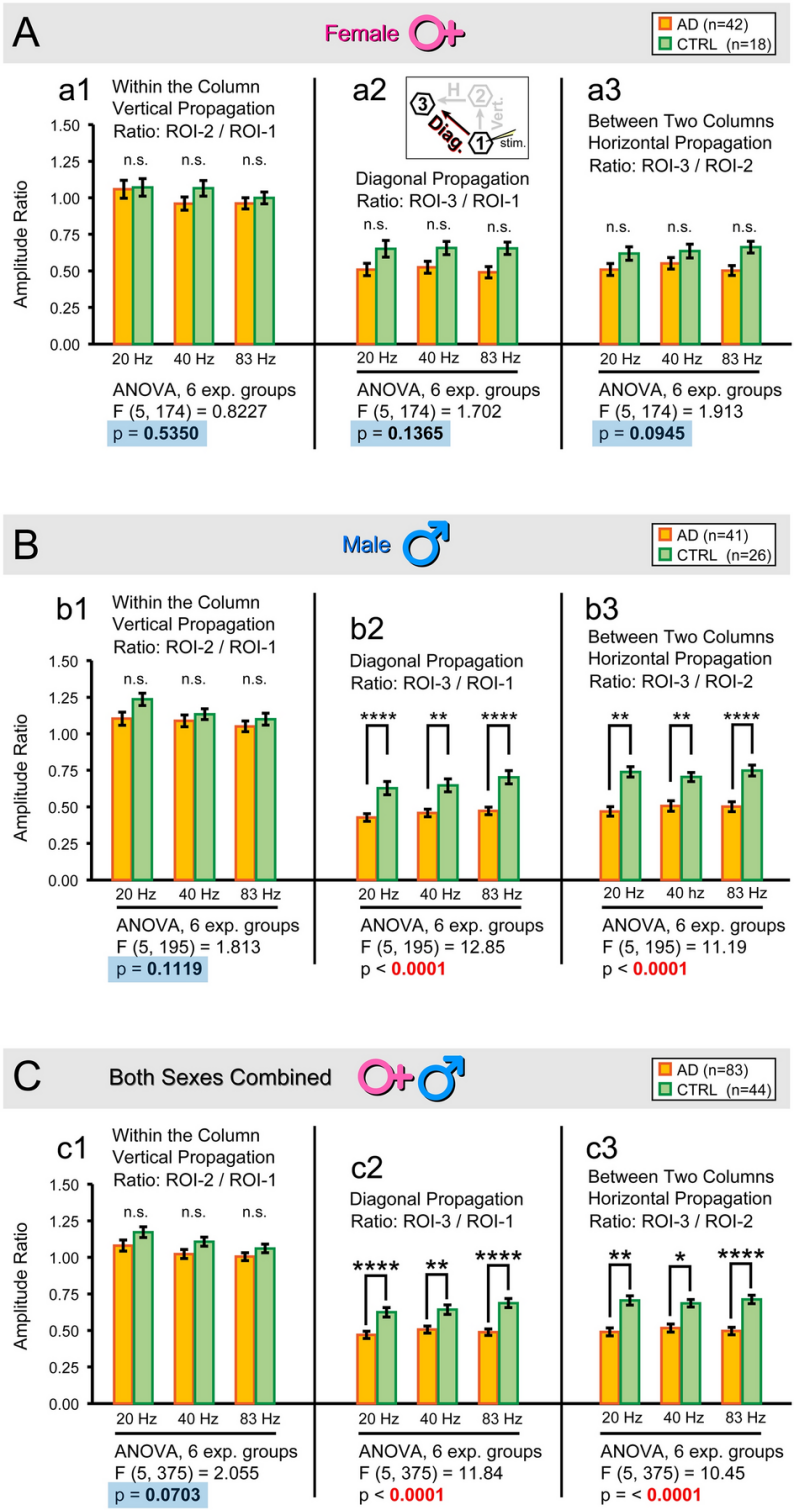
Efficacy of spatial propagation – amplitude ratio between 2 locations. (**A**) Quantification of the optical recordings. The amplitude ratio (e.g., ROI-3 divided by ROI-2, ROI 3/2), was always obtained within the same brain slice and same recording sweep. Each bar represents the average of all slices in the experimental group. Data were obtained from female mice only. The AD-Female group (orange) includes 42 brain slices from 14 mice. The CTRL-Female group (green) includes 18 brain slices from 7 mice. Error bars represent standard error of the mean (s.e.m.). An ANOVA was conducted on 6 experimental groups to compare the effect of genes (AD vs CTRL) on each direction of signal propagation: (a1) *Vertical* direction; (a2) *Diagonal* direction; and (a3) *Horizontal* direction. The meaning of the propagation direction is illustrated Fig. 4A. “Diagonal direction” is again illustrated in the a2-Inset. The ANOVA details are stated below the graph bars, including the F-value and *p* value. The significance of the post-hoc comparisons with a Tukey’s test: [*] p < 0.05; [**] p < 0.01; [***] p < 0.001; and [****] *p* < 0.0001. A bluish background rectangle behind the ANOVA’s *p* value indicates that physiological differences between AD vs CTRL conditions were not statistically significant (the null hypothesis is not rejected). (**B**) Same organization as in (*A*), but data from male mice. The AD-Male group (orange) includes 41 brain slices from 12 mice. The CTRL-Male group (green) comprises 26 brain slices from 8 mice. (**C**) Combined data from both sexes. Note that when sexes were analyzed separately (panels A and B), or combined (panel C), each time the “*Vertical*” voltage signal propagation from ROI-1 to ROI-2 (between layers of the input cortical column), were not significantly affected by the experimental condition (AD vs CTRL), indicated by “*n.s.*”.

We tested if the efficacy of spatial propagation is affected by the presence of AD genes. Simultaneous multi-site measurements of evoked cortical depolarizations allowed us to explore the amount of depolarization that “travels” from the stimulation site (ROI-1) to the remote sites ROI-2 and ROI-3. The schematic of the experiment, stimulation site, and ROI distribution are shown in Fig. 4A. We quantified “*propagation efficacy*” as the ratio of depolarization between two recording locations. For example, the amplitude ratio ROI-2/ROI-1 provides information about the efficacy of propagation from ROI-1 to ROI-2. The ratio ROI-2/ROI-1 represents vertical-propagation within the same cortical column, between L4 and L2/3. A smaller ratio indicates a stronger amplitude decline between L4 and L2/3. Likewise, the ratio ROI-3/ROI-2 represents a horizontal-propagation between two neighboring columns, as depicted schematically in Fig. 4A (horizontal full arrow). Finally, the ratio ROI-3/RO-1 represents the amount of depolarization originating at ROI-1 (the stimulation site) that can reach ROI-3 by signal traveling through layers and cortical columns concurrently (Fig. 4A, dashed oblique arrow). The calculated ratios (ROI 3/2, 2/1, and 3/1) were organized in the graphical display on the basis of: (i) experimental condition (AD vs CTRL), and (ii) stimulation frequency (20, 40 and 83 Hz) (Fig. 6).

In female mice, no statistically significant differences were observed between the AD and CTRL groups at any stimulation frequency—20 Hz, 40 Hz, or 83 Hz—regardless of the propagation direction (ROI 3/2, ROI 2/1, or ROI 3/1) (Fig. 6A, Female 20 Hz, 40 Hz, and 83 Hz) (ANOVA, *p* > 0.05). Although the propagation strength was often slightly greater in the CTRL group (green bars), the differences between AD (orange) and CTRL (green) groups were not statistically significant (post-hoc Tukey’s tests, *p* > 0.05).

### Strength of propagation in the AD-male group

In the male cohort, vertical propagation from L4 to L2/3 of the input column (ratio ROI 2/1) was not significantly affected by AD genes at any stimulation frequency tested (Fig. 6B–b1) (ANOVA, *p* = 0.1119). Across all frequencies (20 Hz, 40 Hz, and 83 Hz), a similar level of depolarization reached ROI-2 in both AD (orange bar) and CTRL (green bar) groups (Fig. 6B, ratio ROI 2/1, n.s.) (post-hoc Tukey’s, *p* > 0.05).

In contrast, significant differences between the AD and CTRL groups were observed in the noncanonical (diagonal) propagation between ROI-1 and ROI-3 at all three stimulation frequencies (20, 40, and 83 Hz) (Fig. 6B –b2 ROI 3/1) (ANOVA, *p* < 0.0001) (post-hoc Tukey’s, *p* < 0.01). Significant differences between the AD and CTRL groups were also observed in horizontal propagation, along the superficial cortical lamina (Fig. 6B –b3) ROI 3/2) (ANOVA, *p* < 0.0001) (post-hoc Tukey’s, *p* < 0.01). Thus, in both the diagonal (ratio ROI 3/1) and horizontal (ratio ROI 3/2) directions, the presence of AD genes reduced the propagation efficacy of electrical signals through the cortical neuropil.

### Strength of propagation in both sexes combined

Combining data from both sexes (Fig. 6C), revealed a weaker propagation efficacy in AD vs CTRL group at each frequency tested, if the ROI-3 (the most remote recording site) was a part of the ratio (e.g., ratio ROI 3/2 and ratio ROI 3/1). Specifically, the vertical direction of propagation (from ROI-1 to ROI-2) was similar between two experimental groups (AD vs CTRL), but the two propagation directions involving the most remote recording site (ROI-3), namely “Diagonal” and “Horizontal”, showed statistically significant differences between AD vs CTRL (Fig. 6C, c2 and c3).

This is likely due to the nature of voltage signal in ROI-3. In the most remote recording site, ROI-3 (Fig. 4A), the contribution of electrical signals caused by direct stimulation of excitable membranes (e.g., action potentials) is the smallest. In ROI-3, synaptic potentials (e.g., EPSPs) contribute more to the optical signal amplitude than in the other two recording sites, ROI-2 and ROI-1, hence a greater chance for AD-induced synaptic dysfunction to reveal itself in the voltage imaging records at this remote location.

Vertical propagation along the same input column ROI 2/1 remained similar between AD vs CTRL, at each frequency tested. Both experimental conditions, AD and CTRL propagate electrical signals vertically (radially) towards pia with identical propensity (Fig. 6C, ROI 2/1, n.s.). This is likely caused by the vertical (radial) orientation of axons and dendrites of pyramidal neurons. The same neuron may have its neurites in two locations, ROI-1, and ROI-2, as illustrated in the cartoon (Fig. 4A). Potential AD vs CTRL differences in synaptic transmission from ROI-1 to ROI-2 would be obscured by optical signals representing direct propagation of voltage transients through the neuronal axo-dendritic tree.

In summary, the current ex vivo voltage imaging data indicate that synaptically-evoked cortical depolarizations propagate through cortical neuropil less efficiently in AD mice compared to CTRL mice. Statistically significant differences emerge only when the most distal recording location, ROI-3, is used to quantify propagation strength (ROI 3/2 or ROI 3/1).

Interestingly, similar amplitude ratios were observed across the three stimulation frequencies tested (Fig. 6C). For example, when propagating from ROI-2 to ROI-3, the signal amplitude at ROI-3 is approximately 50% of that at ROI-2. Specifically, in the AD group, the ROI 3/2 ratio is consistently ~ 0.5, regardless of stimulation frequency (Fig. 6C–c3). In the CTRL group, the ROI 3/2 ratio is consistently ~ 0.6 across all frequencies (Fig. 6C–c3). Regardless of the propagation direction (e.g., diagonal, horizontal), the propagation strength, assessed as the amplitude ratio between two locations on the same brain slice, was uniform across the three frequencies tested—no significant differences were found between 20, 40, and 83 Hz (ANOVA, *p* > 0.05; post-hoc Tukey’s, *p* > 0.05) (Suppl. Fig. S2).

This finding, along with the robust AD-to-CTRL differences observed at all three frequencies (Fig. 6C -c3 and 6C–c3), suggests that stimulation frequency does not significantly alter the propagation of cortical depolarization through the cortical neuropil. In conclusion, simultaneous recordings of evoked compound EPSPs across multiple cortical layers (Figs. 3, 4, 5, 6) revealed no unique properties of the 40 Hz input frequency, which is the only frequency shown to alleviate AD symptoms in AD mice^8,9,21,22^.

## Discussion

We created a mouse line, termed "5xFAD-GEVI", by combining transgenic chi-VSFP mice (GEVI) with 5xFAD mice (AD). This mouse line expresses the GEVI variant "chi-VSFP"^23^ in all cortical pyramidal neurons (Fig. 1A)^10,16–18^ and develops amyloid plaques in the cerebral cortex (Fig. 1BC). To investigate physiological differences between the AD mouse model (experimental group AD, 5xFAD-positive) and their healthy littermates (experimental group CTRL, 5xFAD-negative), we conducted experiments involving extracellular stimulation in cortical layer L4, and multi-site GEVI voltage imaging ex vivo (Fig. 2). The goal was to determine how ex vivo cortical circuit responds to three frequencies of stimulation: *medium-gamma* (40 Hz), *below-gamma* (20 Hz), and *high-gamma* (83 Hz) (Fig. 3C).

### AVV-free approach

Traditionally, in AD model mice, neurons are labeled with fluorescent indicators (e.g., GCaMP6) through the injection of brains with viral vectors (AAV)^24–26^. In our study, we opted for an AAV-free approach and instead adopted a transgenic animal strategy. Transgenic strategy offers several advantages: it is more robust and repeatable, provides uniform labeling across wide cortical areas (wide cortical areas are uniformly labeled Fig. 1A), reduces experimental variations, and is less labor intensive compared to the AAV strategy.

### Population voltage imaging and compound EPSPs

Population voltage imaging lacks "single-cell resolution", as it aggregates neuronal circuit activity from a group of cells into a compound signal. Within the dye-stained or GEVI-labeled region of the neocortex, the largest percentage of membrane area is contributed by the dendrites of pyramidal neurons. Consequently, the voltage-imaging signal is primarily influenced by dendritic potentials^27,28^. Multiple lines of evidence suggest that the population voltage signal mainly consists of EPSPs occurring in dendrites^29–31^, similar to the local field potential (LFP)^20^. This stands in stark contrast to calcium imaging methods or single-unit electrode recordings, which predominantly detect neuronal spiking (action potentials).

### Similar voltage waveforms at multiple recording sites

In our working model, we assumed that the number of dendrites and cell bodies directly activated by the stimulation electrode field (through direct depolarization of the membrane by a brief stimulus pulse) was significantly greater at the stimulation site (ROI-1) compared to the most remote site (ROI-3). Consequently, we expected the optical signal at ROI-1 to have significantly greater contributions from direct depolarizations and action potentials (APs) than at ROI-3, where the optical signal is primarily driven by synaptic release. Depolarization at ROI-3 would only occur if axons of neurons activated at ROI-1 invade ROI-3 and release glutamate. Given this model, we must consider whether the visually similar voltage waveforms recorded at the three ROIs (Fig. 5GH) contradict our assumptions.

The similar voltage waveforms across ROI-1, ROI-2, and ROI-3 are likely due to the minimal impact of direct depolarizations (1-ms stimulus pulse) and APs (1–2 ms duration) on the optical signal. Instead, the shape of the optical signal is overwhelmingly dominated by slower synaptic potentials, which overlap in the hundreds of dendritic branches captured within each ROI^29–31^.

Despite the relatively similar waveform shapes observed at the three locations (Fig. 5GH), three lines of our experimental evidence support our initial premise that the number of dendrites and cell bodies directly activated by the stimulation electrode’s field is indeed greater at the stimulation site (ROI-1) than at the most remote site (ROI-3):

1. The amplitude of the maximal evoked optical signal (Max P) decreases with increasing distance from the stimulation site (Fig. 4F).
2. The amplitude ratio between the more distal (ROI-3) and more proximal locations (ROI-2) is consistently and substantially smaller than 1.0, across all data sets, female, male, and combined (Fig. 6, ROI-3/ROI-2).
3. Temporal summation efficacy, driven by the summation of EPSPs, increases with distance from the stimulation site (Suppl. Figure 1A, B).

### Medium-gamma (40 Hz) assumed mechanism

It has been shown that an invasive artificial stimulation of cortical circuits at the *medium-gamma* (40 Hz), but not other frequencies, recruits neuronal and glial responses to reduce Aβ levels in the visual cortex^21^. Combining *medium-gamma* pulses of both light and sound has demonstrated protective effects on the visual, auditory, hippocampal, and prefrontal cortices in AD model animals, leading to improvements in spatial and recognition memory, as well as synaptic function^8,9,22^. The assumed mechanism underlying these effects is the restoration of Alzheimer’s disease-altered oscillations in brain networks^7,9,32^ and promotion of glymphatic clearance of amyloid^33^. However, Soula et al. reported that 40-Hz flickering simulation did not engage native gamma oscillations in the visual cortex, entorhinal cortex, or the hippocampus. Furthermore, the AD pathology was not altered by the 40-Hz sensory stimulation^34^. Soula et al. cautioned that failure to observe robust changes in AD pathology after short-term or chronic sensory exposure^34–37^ should not be taken as final evidence against potential beneficial outcome in mice or humans chronically exposed to combined 40-Hz flickering light and sound stimulation^8,9,38–40^. Since the exact mechanism of the 40-Hz stimulation remains intriguing, we developed a hypothesis that at a 40 Hz input frequency, subthreshold synaptic events undergo (i) optimal temporal summation, or (ii) optimal spatial propagation through the local cortical circuit. To test this hypothesis, we delivered a train of 10 synaptic stimuli (40 Hz) into L4, the primary input layer of the neocortex^41^. In the same brain slice, same recording sites (ROIs) we also tested synaptic trains using lower (20 Hz) and higher frequency (83 Hz), for comparison (Figs. 3, 4, 5, 6).

### Temporal summation efficacy

At higher stimulation frequencies (40 Hz and 83 Hz), temporal summation (paired pulse ratio, P2/P1) demonstrated greater strength at the most remote site (ROI-3) compared to the proximal stimulation site, ROI-1 (Suppl. Fig. S1). This disparity is attributed to the nature of the optical signal at the three ROIs (Fig. 4A). Action potentials (APs), which contribute minimally to temporal summation as they do not summate, are more abundant at the stimulation site (ROI-1). Consequently, temporal summation at ROI-1 often exhibited lower efficacy than at the remote sites (ROI-2 or ROI-3), where the synaptic contribution to the optical signal was more pronounced.

### Strength of the voltage signal propagation

Our simultaneous multi-site measurements, conducted in two cortical layers (L5 and L2/3), have revealed no change in evoked responses between ROI-1 (located in L5) and ROI-2 (located in L2/3). The propagation from ROI-1 to ROI-2 occurs through the apical dendritic branches of cortical pyramidal neurons via electrotonic processes. In contrast, propagation from ROI-2 (within L2/3 of the input cortical column) to ROI-3 (within L2/3 of the neighboring cortical column) occurs via axons and synapses. This suggests that any changes detected in the non-canonical (*diagonal*) direction, from ROI-1 to ROI-3, are primarily due to alterations in the horizontal, intralaminar propagation from ROI-2 to ROI-3. This would imply that only the propagation between ROI 2 and 3 is altered by the AD genes. In the Female cohort (Fig. 6A), Male cohort (Fig. 6B), and both sexes combined (Fig. 6C), the R3/1 and R3/2 ratios are not only consistently higher in CTRL compared to the AD genotype, but also achieve similar values—~ 0,6 in the CTRL, and ~ 0.5 in the AD cohort.

To summarize, in both sexes, the AD pathology led to a reduction in the strength of compound EPSP voltage signal propagation through cortical neuropil. In both female (Fig. 6A) and male (Fig. 6B) cohorts, the green bars (CTRL) were consistently higher than the orange bars (AD). However, notable (statistically significant) AD vs CTRL distinctions surfaced only in the male cohort (Fig. 6B, asterisks), but were also present in the *Both Sexes Combined* cohort (Fig. 6C, asterisks). Based on the analyses in this specific 5xFAD-GEVI mouse model, we have not found any evidence that 40-Hz stimulation improves the spatial propagation of cortical depolarizations between layers, or between cortical columns.

## Supplementary Information

Supplementary Information.

## Data Availability

The datasets generated during and/or analyzed during the current study are available from the corresponding author on reasonable request.
