# Supplementary material for: Ex vivo propagation of synaptically-evoked cortical depolarizations in a mouse model of Alzheimer’s disease at 20 Hz, 40 Hz, or 83 Hz
Source: Sci Rep. 2024 Oct 8;14:23365. doi: 10.1038/s41598-024-74262-2 (PMC11458755; doi:10.1038/s41598-024-74262-2)
Supplement: Supplementary file 1 — Supplementary Information. [file 41598_2024_74262_MOESM1_ESM.pdf]

Suppl. Fig. 1

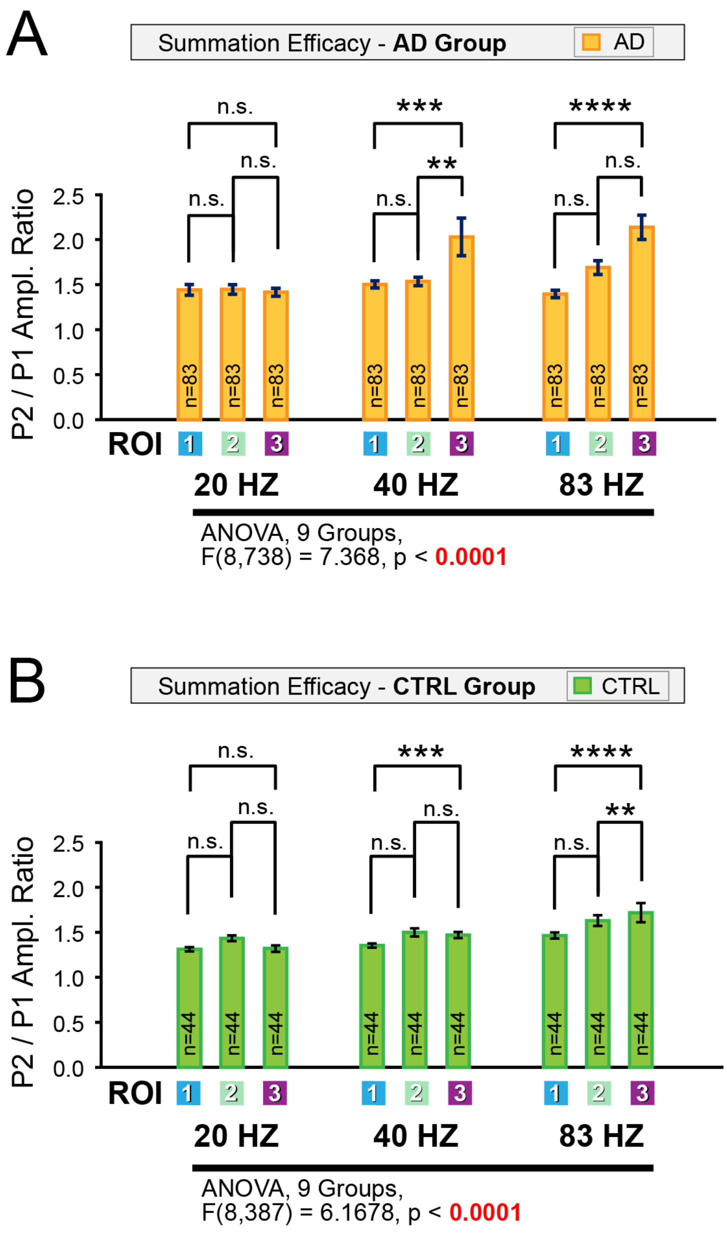

Suppl. Fig. 1. Efficacy of Temporal Summation (Peak-2 / Peak-1 Ratio) Varies with Distance from the Stimulation Site.

A) At higher stimulation frequencies, specifically 40 Hz and 83 Hz, post-hoc Tukey's tests revealed statistically significant differences between ROI-1 (the stimulation site) and ROI-3 (the most distal recording site). The locations of the ROIs on the surface of the brain slice are shown in the main figure, ***Fig. 4A***.

B) A similar pattern was observed in the CTRL mouse cohort. The number of brain slices per group is indicated by "n". The significance of post-hoc comparisons using Tukey's test is denoted as follows: ["n.s."] p>0.05; [*] p<0.05; [**] p<0.01; [***] p<0.001; and [****] p<0.0001.

++

Suppl. Fig. 2

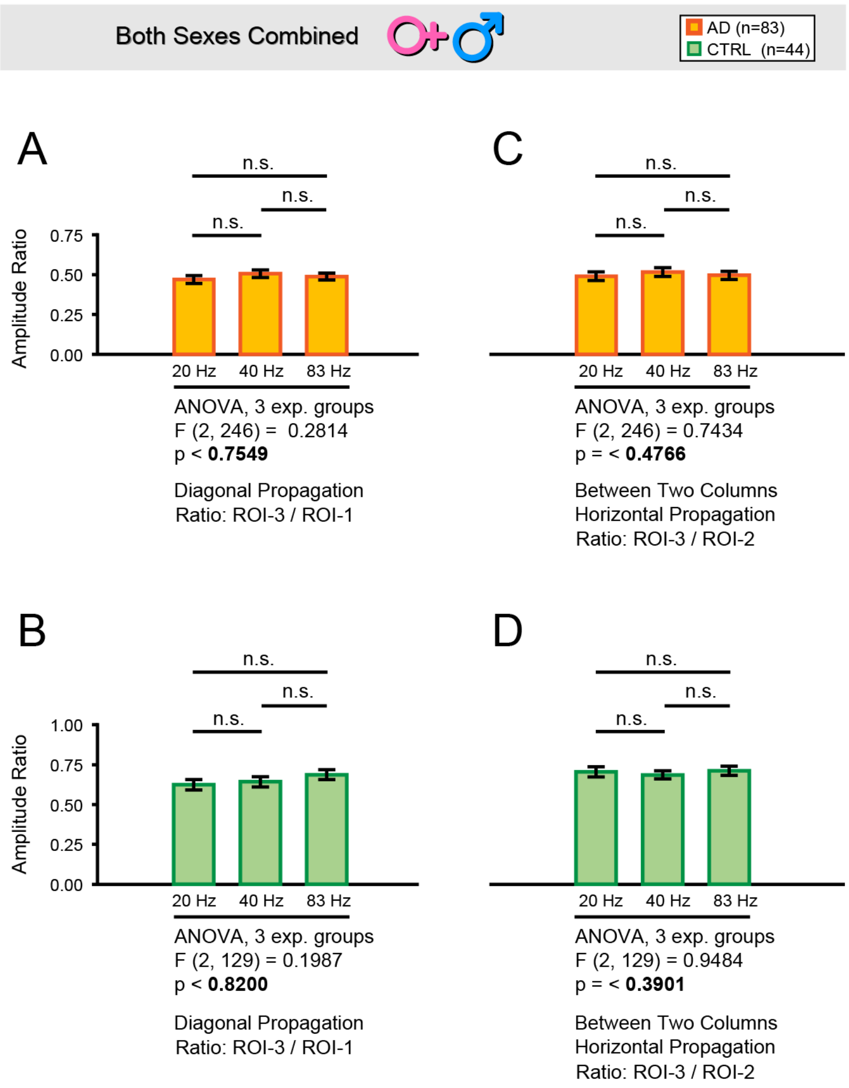

Suppl. Fig. 2. The Efficacy of Spatial Propagation is Independent of Synaptic Stimulation Frequency.

A) As illustrated in ***Fig. 4A***, the diagonal propagation refers to the synaptically-evoked cortical depolarizations traveling from the stimulation site (ROI-1) to the most remote recording site (ROI-3). The strength of this "*diagonal propagation*" is quantified by the amplitude ratio of the optical signals recorded at ROI-3 and ROI-1 (ROI-3 / ROI-1). Post-hoc Tukey's tests showed no statistically significant differences in this ratio across the three stimulation frequencies used in the experiments (20, 40, and 83 Hz).

B) Similar results were observed in the CTRL mice cohort, with no significant differences in the ROI-3 / ROI-1 ratio across the three stimulation frequencies.

C) Likewise, horizontal propagation, measured from ROI-2 to ROI-3, did not show statistically significant differences across the three stimulation frequencies (20, 40, and 83 Hz).

D) Post-hoc Tukey's tests confirmed that the ROI-3 / ROI-2 ratio (representing horizontal propagation) was not significantly different among the three stimulation frequencies.

++
